# Highlights of the ‘I Congress ecancer Choosing Wisely’ March 30 and 31, 2022 Santa Cruz, Bolivia

**DOI:** 10.3332/ecancer.2022.1436

**Published:** 2022-08-04

**Authors:** Ronald Limón, Lucia Reynolds, Erick Rocha, Lucia Richter, Mario Gianella, Oscar Niño de Guzman, Gerson Mejia, José Nina, Wendy Rojas, Lijia Avilés, Iván Maldonado, Cristian Pacheco, Claudio Martín, Maria G Cervantes, Federico Bakal, Eduardo Cazap

**Affiliations:** 1 Department of Oncology, University Social Security USS, Nº58 Colon Street, 10260 Santa Cruz, Bolivia; 2 Department of Hematology and Oncology, Clinic of the Americas Santa Cruz, Bolivia Nº 5100 Beni Av, Santa Cruz, Bolivia; 3 Department of Oncology and Research, Martin Dockweiler University Hospital, Nº1 Street A, 3rd External Ring Avenue, Santa Cruz, Bolivia; 4 Department of Oncology, OncoBolivia Specialized Center for Cancer Treatment, Nº236 Azucenas St Equipetrol, Santa Cruz, Bolivia; 5 Department of Oncology, Health Oil Box Hospital, Nº 3 Rafael Peña St, Santa Cruz, Bolivia; 6 Department of Surgery, Niño de Guzmán Gynecology and Oncology Specialized Center for Cancer Treatment, Nº564 Oquendo Av, Cochabamba, Bolivia; 7 Department of Oncology, Clinical Hospital Viedma, Venezuela St, Cochabamba, Bolivia; 8 Department of Oncology, Hospital of the National Health Fund, La Paz, Bolivia; 9 Relieve Pain Treatment Center, Nº56 Barasea St, Santa Cruz, Bolivia; 10 Oncoservice Radiotherapy and Nuclear Medicine Center, Nº13 Hernán Síles Av, La Paz, Bolivia; 11 OncoHope Cancer Treatment Center, Nº 143 Eloy Alfaro Av, 170147 Quito, Ecuador; 12 Department of Clinical Oncology, National Institute of Neoplastic Diseases, Nº2520 East Angamos Av, Lima, Peru; 13 Department of Thoracic Oncology, Alexander Fleming Institute, Nº 1180 Crámer St, 1406 Buenos Aires, Argentina; 14 Department of Oncology, National Medical Center 20 Noviembre, ISSSTE, Nº 540 Félix Cuevas St, 03229 Mexico City, Mexico; 15 Department of Radiotherapy, Oncology Institute, Fundación Arturo Lopez Perez, Nº805 José Manuel Infante St, 1030000 Santiago, Chile; 16 Latin American and Caribbean Society of Medical Oncology (SLACOM), Buenos Aires, Argentina

**Keywords:** choosing wisely, oncological surgery, palliative care, clinical oncology

## Abstract

The ecancer ‘Choosing Wisely’ conference was held for the first time in Latin America in Santa Cruz, Bolivia. The event had more than 150 registered attendees in addition to 22 speakers from different countries and different specialities in the field of oncology, who presented topics on prevention, oncological surgery, clinical oncology and palliative care, in order to demonstrate the current evidence of how to approach a patient in daily clinical practice based on the human resources, materials and drugs available, trying to offer the maximum benefit to the patient based on current scientific evidence. In addition to addressing issues of vital importance in breast cancer, during the 2 days of the event, updated information generated in recent years was presented, the results of which will change clinical practice. All the experts were in favour of developing strategies and methods that help us to properly select treatments to optimise resources and reduce the economic toxicity of the most modern and current treatments. This conference was an event of vital importance because it was the first face-to-face event for ecancer and the physicians after difficult years due to COVID-19.

## Introduction

The COVID-19 pandemic that began in 2019 was an event that forced us to look for new ways to share updated medical information. In this case, virtuality was the method that had the most impact to meet the objectives. Returning to face-to-face events and being the first Latin Americans to resume this modality was of vital importance for the event, since we believe that despite the advantages of virtuality, person-to-person interaction is important for the exchange of ideas and concepts. Unfortunately, the pandemic greatly affected people with chronic diseases but also, and to a large extent, patients with oncological diseases, producing a negative impact on them. Some of these negative aspects were: 1) delayed detection and diagnosis, 2) delayed administration of oncological therapies and 3) delayed surgical procedures. This will have a long-term negative impact on cancer statistics worldwide where cancer mortality is expected to be higher than in previous years, all due to the pandemic. Another negative effect of the pandemic in the field of oncology was the premature closure of clinical trials and the cancellation of academic events worldwide, which forced us to develop virtual events trying to provide updated information. After difficult moments during the years after 2019, holding a face-to-face event allowed us to share information where attendees could interact with experts, which we believe is of vital importance. The event *e*cancer Choosing Wisely Santa Cruz, Bolivia, was held on March 30 and 31, 2022, addressing for the first time in Bolivia the concepts of choosing wisely in the different areas of oncology. During the first day of the session, topics such as choosing wisely principles, pharmacology, radiotherapy, screening and detection, oncological surgery, precision medicine, pain therapy and oncology interventionism and clinical trials were touched upon. The second day of the conference was dedicated to breast cancer, addressing topics from the San Antonio Breast Cancer Symposium 2021, American Society of Radiotherapy Oncology (ASTRO) 2021 and topics of vital importance in breast cancer such as: surgery in breast cancer in advanced stages, treatment of breast cancer during pregnancy, treatment of male breast cancer, management of breast cancer with relapse only at the level of the central nervous system and neoadjuvant endocrine therapy, among others [2–6].

## Highlights day 1

Dr Eduardo Cazap, President of SLACOM, Argentina, and Editor-in-Chief, *e*cancer, began by giving a definition of the meaning of choosing wisely applied to current medicine, mentioning that the initiative to choose wisely has its origin in the American Board of Internal Medicine created in 1989 and that it defined choosing wisely as the initiative that seeks dialogue to avoid unnecessary medical tests, treatments and procedures in patients, in addition to commenting that the main focus of this initiative is to promote conversation between doctors and patients to help patients choose the best care for them based on the current scientific evidence. He mentioned that this problem affects all patients of the different specialities, not only cancer patients, giving examples that most of the economic and human resources for patient care are generally given during the last 3 months of life, when the treatments are probably less effective or null. He recalled how this Choosing Wisely initiative began in the field of medicine going back to the year 2012 in the United States [1] and that is where it has spread to different parts of the world: Europe, Asia, Oceania, the Middle East and Latin America. As a last message at the end of his talk, he called for reflection that this initiative has a medical component and that it shows us the best evidence and the best clinical practices, but it also has a public health component that involves regional governments and that it is necessary to design policies that avoid unnecessary medical tests and treatments in order to optimise human and economic resources.

Later, Dr Erick Rocha, Bolivia, spoke about choosing wisely in oncological surgery, saying that choosing an oncological procedure wisely leads to better results and improvements in the patient’s quality of life, taking as an example a patient with rectal cancer in the early stages where minimally invasive transanal surgery represents the current standard of care in this pathology, arguing that approximately 30% of cases occur at the level of the lower rectum where applying this procedure is safe for the patient and provides excellent results, to close his talk he mentioned that this technique is a clear example of how choosing wisely allows us to perform less invasive procedures compared to conventional surgery.

Next, Dr Claudio Martin from the Alexander Fleming Institute, Buenos Aires, Argentina, spoke about precision medicine. He talked about the concept of What is precision oncology? He defined precision medicine as drugs oriented to a specific molecular target independent of the location of the tumour; the ultimate goal of precision medicine is to find a drug for a specific alteration. In recent years, we have had very important advances in medicine; taking lung cancer as an example, advances in precision medicine in lung cancer, a deadly disease until very recently, have made it possible to see changes in specific mortality from this disease. In 2019, approximately 20% of patients with lung cancer were detected with some molecular alteration that could be treatable at that time, thanks to the progress of the tumour genome, we can currently detect 45% with some molecular alteration and trigger this mutation to produce a therapeutic effect. Different techniques are available that have allowed us to identify these molecular alterations either from tumour tissue or through blood, which we now know as liquid biopsy, in addition to these molecular techniques allowing us to identify resistance variations or mutations after a previous treatment. Thanks to this, we can identify them early and this allows us to change specific treatment if necessary. In fact, the choice of treatment will depend on the benefit that can be offered to the patient and the access to these drugs.

Regarding radiotherapy, Dr Federico Bakal, Fundación Arturo López Pérez (FALP), Chile, began his presentation by referring to an article published in 2008 in the United States, which mentioned health costs [7]. Something particular about this study was that the results were not very different compared to other countries; however, the costs for medical treatments in the United States were higher without showing a greater benefit in terms of results related to mortality. During his talk, he made reference to our medical training since many of these points have to do with our training; that is, to what extent do we, as doctors in training and then as specialists, need to request tests? At what point should we stop treating patients? These are questions that to this day do not have an answer.

Dr Bakal said that many factors influence decision-making; for example, The ASTRO includes recommendations on when we should stop or when we should not give radiation to the patient and there are some that we describe below because it indirectly recommends that we should choose wisely when, how and to whom we should treat:

- Prostate cancer: 1) Do not give proton radiation outside of clinical trials. 2) Do not give radiation to low-risk patients without discussing the benefits of active follow-up.
- Breast cancer: 1) Do not routinely offer intensity-modulated radiation therapy or whole-brain radiation therapy (WBRT) in conservative surgery. 2) Do not start treatment without considering shorter fractionations. 3) Do not perform more frequent mammograms due to conservative surgery.
- Lung Cancer: 1) Do not perform adjuvant radiotherapy in patients with N0-1 lung cancer.
- Endometrial cancer: 1) Do not offer radiotherapy to patients with low-risk endometrial cancer.
- Palliative radiotherapy: 1) Do not start palliative radiotherapy without talking to the patient about its benefits, and having previously referred the patient to palliative care. 2) Do not supplement WBRT post stereotactic radiosurgery. 3) Do not perform more than ten fractions for bone metastases. It is important to consider all the clinical and pathological aspects of the patient in order to offer the best available treatment, optimising our resources. This is a great example of how choosing wisely can be applied in all fields of medicine and that it is something that we have been indirectly applying already.

Closing the first day, there was a presentation by Dr Carlos Castro, director of the Colombian League Against Cancer, from Colombia, who referred to how human papilloma virus (HPV) vaccination has dramatically changed the fight against cervical cancer. We know the importance of getting vaccinated against HPV because it is definitely a debt that we doctors owe to our population. Statistical data shows that 85% of cervical cancer deaths occur in low to middle income countries – within these many Latin American countries. We must remember that cervical cancer is detected mostly between 35 and 45 years of age with a median age of 50 years at diagnosis.

Dr Castro, a pioneer in cancer prevention, mentioned that the HPV vaccine has favourably changed our fight against this disease since it is safe, effective and is distributed free of charge in Colombia, as in many other Latin American countries. He later referred to the fact that one of the most important barriers that we must combat is misinformation regarding vaccination against this type of virus with oncogenic risk. Finally, he concluded his talk by showing data from Colombia [19] where there is evidence of a decrease in the prevalence of cervical cancer and a significant increase in the vaccination rate as the greatest success factor for these results, in addition to the fact that, thanks to current research, a dose of the HPV vaccine would be enough to reduce the risk of developing cervical cancer.

## Highlights day 2

The second day was dedicated to the treatment of breast cancer, addressing issues generated during recent congresses such as ASTRO and the San Antonio Breast Cancer Symposium (SABCS).

Niño de Guzmán MD (Niño de Guzmán Gynecology and Oncology Specialized Center for Cancer Treatment, Bolivia) opened the day by talking about three important studies presented at SABCS 2021, the first of which was the SINODAR-ONE study, a prospective multicentre randomised non-inferiority study aimed at evaluating the therapeutic role of axillary lymph node dissection (ALND) in patients undergoing breast cancer surgery (BCS) or mastectomy for T1-2 breast cancer (BC) presenting 1–2 macrometastatic sentinel lymph nodes (SLNs); the characteristics of the patients were: age > 40 and < 75 years, breast carcinoma, unifocal-unilateral lesion, size < 5 cm, axillary nodes clinically negative (N0), no more than two sentinel nodes with macrometastases, no distant metastases, no neoadjuvant treatment, no prior breast cancer; patient stratification was 443 patients to control arm, of whom 417 underwent axillary dissection radical and of 440 in the experimental arm, only 13 patients underwent radical axillary dissection. The results did not show a statistically significant difference in terms of recurrence-free survival or overall survival at the time of the outcome evaluation cutoff.

The second study was Patient Reported Outcomes for the Intergroup Sentinel Breast Study (INSEMA, GBG75, ABCSG43): Persistent impact of axillary surgery on arm and breast symptoms in early breast cancer. The main objective is to compare invasive disease free survival (iDFS) after BCS (noninferiority question) between patients without axillary surgery and sentinel lymph node biopsy (SLNB) (first randomisation). The key secondary endpoint was to compare iDFS after BCS (noninferiority question) between SLNB alone and patients with complete ALND (c) (second randomisation). Another very important secondary endpoint was quality of life (QoL). INSEMA (involving over 5,000 patients) is one of the first randomised trials investigating SLNB omission in clinically node-negative patients and the first to report quality of life data. The results showed that patients without SLNB had improved breast and arm symptoms compared to those with SLNB; another result was that patients in the SLNB group had improved symptoms and arm function compared to those receiving ALND. No relevant differences were observed in the other QoL scales.

The iDFS data (main result) is expected to be presented at the end of 2024 and lastly Dr Niño de Guzmán referred to the study that evaluated the *Impact of Race and Ethnicity on the Incidence and Severity of Lymphedema Related to Breast Cancer After Axillary Lymph Node Dissection: Results of A Prospective Study* [8]. Patient recruitment was between November 2016 and March 2020; patients were women older than 18 years with post-ALND and SLNB breast cancer. The results showed a high incidence of oedema in black women undergoing ALND and radiotherapy; however, neoadjuvant chemotherapy (NAC) increased the risk of lymphoedema compared to adjuvant therapy in women undergoing ALND and radiotherapy. The mechanism by which NAC increases the incidence of lymphoedema is unknown, it is hypothesised that NAC could cause fibrosis of tumour-filled lymphatic vessels as well as lymphatic endothelial damage before surgery, resulting in lower rate discharges of lymphoedema after ALND. As it is important to individualise each case, for example, subgroups of patients unlikely to achieve nodal pathological complete response (pCR) (e.g. HR+/HER2−), alternatives to NAC are needed to prevent ALND.

The following talk was dedicated to the treatment of breast cancer in men, which was given by Cristian Pacheco MD (National Institute of Neoplastic Diseases, Peru), who referred to the fact that this type of cancer represents less than 1% of cancers in men and in a ratio of 1 to 8 in relation to breast cancer in women, the median age of presentation is around 65 years, there are clearly risk factors such as age, hormonal imbalance (androgen – testosterone), Klinefelter syndrome, exogenous oestrogens, chest radiation, family history of cancer, genetic mutations in relation to genetic-hereditary cancer such as BRCA1-2, PLAB2, CHECK2, PTEN [9]. Regarding epidemiology, the literature reports that in Africa, breast cancer is present in between 5% and 15% of the population, a high number in relation to the rest of the world. Breast cancer in men is usually unilateral and advanced, 90% of cases correspond to ductal histology and with high expression of HR (ER/PgR), in some cases this disease can present synchronously, that is, together with other types of cancer such as prostate, colon and pancreas.

Regarding the treatment of this pathology in both men and women, it is a multimodal treatment, which includes surgery, chemotherapy, hormonal therapy and radiotherapy. It is important to take certain considerations, generally the surgical treatment of choice is total mastectomy, while hormonal therapy is tamoxifen. When choosing a hormonal treatment, initial aromatase inhibitors are not recommended since these drugs produce an increase in secretion of androgens due to failure in the feedback of gonadotropin secretion. We must also consider that 20% of oestrogens are produced in the testis, these oestrogens would not be inhibited by aromatase inhibitors since they do not have action at the gonadal level, that is, it is important that all patients with this type of disease have timely and adequate genetic evaluation and counselling in order to carry out adequate genetic tests, since depending on the gene found, the risk of breast cancer in men could reach up to 6.8%.

Finally, breast cancer in men is a rare disease; however, the advances in the diagnosis and the biology of the disease have allowed us to develop treatment strategies similar to breast cancer in women. Regarding the use of cyclin inhibitors, their use is recommended in metastatic disease after a first line of treatment has failed, and currently, all clinical trials admit male patients with breast cancer who meet the inclusion criteria in order to generate adequate scientific information for its safe use.

Next, María Guadalupe Cervantes MD (National Medical Center 20 Noviembre, ISSSTE, Mexico) gave us a keynote speech on the treatment of breast cancer during pregnancy. She said that pregnancy-associated breast cancer has not only a medical component, but also an ethical, social, psychological component, etc. Breast cancer during pregnancy is defined as cancer that affects a woman during pregnancy or in a period of 12 months after childbirth, and some authors define this disease as cancer that affects a woman from pregnancy to the end of life or the end of breast-feeding. Thanks to medical information, we can see that this disease has a small increase from 30 years of age and from 40 years onwards. Regarding the histological types, we can say that ductal histology is the most frequent with 70%–95%, they are generally high-grade tumours, with a greater possibility of developing axillary lymph node infiltration and the predominant molecular subtype is triple negative breast cancer (TNBC) in approximately 40%, due to the growth of breast tissue due to the hormonal effect produced by pregnancy, most tumours are locally advanced at the time of diagnosis, there are some radiological studies necessary for staging, such as: 1) Ultrasonography, 2) Magnetic Resonance without contrast, 3) Mastography only if necessary and 4) Chest X-ray with foetal protection and there are other studies that are completely contraindicated such as: bone scan, tomography and PET/CT.

Dr Maria Guadalupe then raised a very important question: Should the pregnancy be terminated? Multiple meta-analyses show that the termination of pregnancy has no impact on overall survival and progression-free survival in pregnant women with breast cancer. Regarding treatment, she stressed that treatment should be multidisciplinary and foetal surveillance should be available every 3 to 4 weeks and/or prior to each cycle of chemotherapy. For the therapeutic decision, we must first identify in which trimester of pregnancy the patient is, since the behaviour could change accordingly since a woman with breast cancer during the first trimester of pregnancy is not the same compared to women who have this disease during the second or third trimester of pregnancy.

Axillary treatment and especially sentinel lymph node study is recommended with technetium 99, and, generally, this procedure is performed only during the third trimester of pregnancy. Speaking a little about local treatment, Dr Guadalupe said the consensus on breast cancer during pregnancy recommends mastectomy especially during the first trimester of pregnancy and that for obvious reasons because radiotherapy is contraindicated throughout the pregnancy process, indeed there are exceptions to this and that is if a woman is towards the last part of her pregnancy, conservative surgery associated with radiotherapy could be considered but as we mentioned earlier, this must be agreed upon by the entire multidisciplinary team.

Turning to systemic treatment, it should be noted that during the first trimester of pregnancy, 20% could develop foetal malformations thanks to the use of chemotherapy, and the most frequently used drugs such as anthracyclines showed that they could generate complications in up to 4%–5% of pregnant women patients such as: preterm pregnancies, urethral reflux and equine foot. Regarding the use of taxanes, what was most observed was low weight, growth restriction and preeclampsia. Dr Guadalupe also presented information on other drugs such as cyclophosphamide, which is commonly combined with anthracyclines where it was possible to see that this drug also produces intrauterine growth retardation as the most frequent adverse effect; concluding that the adverse effects of chemotherapy could probably reach 6% of patients who start treatment during the second and third trimesters of pregnancy. Later she referred to more modern drugs, taking trastuzumab as an example, mentioning that in a 2021 publication, trastuzumab interferes with the VGEF signalling that is responsible for the production and reabsorption of amniotic fluid. She mentioned that in this study, approximately 58% of the patients presented oligohydramnios or anhydramnios, so the use of trastuzumab during pregnancy is contraindicated due to the high risk of death due to foetal lung hypoplasia and foetal malformations [10].

Lastly, Dr Guadalupe made reference to the information from the congenital disease society where in her article she showed us how during the first trimester of pregnancy there is a greater risk of developing foetal problems and malformations compared to the second and third trimesters where these problems fall to approximately 4%–6% [11]. Finally, Dr Guadalupe mentioned that there is an increase worldwide in the incidence of breast cancer during pregnancy; she said that there are no changes in treatment standards, and when there are changes for the protection of the fetus, does not recommend the use of targeted antibodies. She said that, in the majority of cases, interruption of pregnancy is not recommended, and finally tells us that the administration of chemotherapy is safe during the second and third trimester of pregnancy.

Next a very controversial topic was discussed which was the role of primary tumour surgery in patients with metastatic breast cancer, this presentation was given by Dr Mario Gianella (Hospital Caja Petrolera de Salud, Santa Cruz, Bolivia) who said that approximately 6%–8% of patients debut with *de novo* metastatic disease, that is, with metastasis at the time of diagnosis, he referred to the fact that there is still no consensus on when to operate and which subtype could benefit most from surgery. During the last 20 years, different retrospective studies have been carried out, which showed some benefit of surgical treatment, however they lacked validity because they were retrospective studies. However, the results of prospective studies by Badwe *et al* [12] in 2017 and Soran *et al* [13] in 2018 show that, respectively, no benefit from primary tumour surgery, and a benefit in overall survival when the primary tumour was operated on; however, these studies had some biases such as suboptimal therapies depending on the subtype of breast cancer.

Finally, in 2020, Seema *et al* [14] presented a randomised clinical trial for patients with metastatic breast cancer whose primary objective was overall survival. It is very important to mention that all patients received optimal systemic therapy depending on histological subtype, a total of 256 were chosen for the study, the majority of patients underwent mastectomy and, in addition, radical armpit surgeries were omitted in the vast majority. Regarding the results, Dr Gianella showed us that there were no differences in overall survival when the primary tumour was operated on, except in the triple negative subtype, where the patients subjected to local treatment had a worse overall survival compared to the control group. Based on the literature presented, he concluded that the study by Dr Seema Khan shows no differences in overall survival except in the triple negative subtype where overall survival was worse with early local treatment, early local treatment can be applied individually since it reduces the risk of local progression and probably the best recommendation for surgery of the primary tumour is when a patient progresses locally during systemic treatment.

Following this was a presentation by Ligia Avilés MD (Oncoservice, Bolivia), where she told us about the highlights of ASTRO 2021, touching on important issues, starting with ultrahypofractionation, radiation throughout the breast, presented by Dr Rachel Rabinovich (University of Colorado Hospital, USA). Dr Ligia Avilés referred to the fact that we currently have enough evidence to use hypofractionation safely and with excellent results. She tells us that hypofractionation also reduces skin toxicity, which is excellent for patients. She commented on the FAST FOWARD study saying that this study probably has the most evidence for hypofractionation plus this treatment allows us to shorten treatment days to only 5 days without compromising the final results compared to other previous studies on hypofractionation and conventional therapy [15, 16].

Finally Ronald Limón MD (University Social Security, Bolivia), told us about two studies presented at SABCS 2021. The first study was the EMERALD study [11], a phase 3 trial on Elacestrant, an oral selective oestrogen receptor degrader (SERD) versus the investigator’s choice of endocrine monotherapy for ER+/HER2− advanced/metastatic BC (mBC) after progression on endocrine and prior CDK4/6 inhibitor therapy. Elacestrant (RAD1901) is an oral SERD that blocks the ER in a dose-dependent manner and inhibits oestradiol-dependent induction of gene transcription and cell proliferation in ER+ BC cell lines. In a phase 1 study of elacestrant in postmenopausal women with ER+/HER2− mBC, single-agent activity was observed at the recommended phase 2 dose (400 mg daily) with confirmed partial responses in highly pretreated patients, including those with prior CDK4/6i and prior fulvestrant, as well as such as those whose tumours harboured mESR1. The study design was men and postmenopausal women with advanced/metastatic, ER-positive, HER2-negative breast cancer, who have progressed on or after 1 or 2 lines of endocrine therapy for advanced disease, one of which was administered in combination with CDK4/6i, ≤ 1 line of chemotherapy for advanced disease ECOG performance status (PS) 0 or 1. Four hundred and forty-seven patients were randomised 1:1, Elacestrant 400 mg daily arm versus Physician’s choice.

The study had two primary endpoints: PFS among all patients (mESR1 and mESR1 undetectable) and among patients with mESR1. The secondary endpoints were: overall survival in all patients and in patients with mESR1 (tested only in case of statistically significant outcome); of PFS in the respective population) to be performed at the time of PFS analysis and when ~50% of patients have died. The study showed that Elacestrant is associated with a 30% reduction in the risk of progression or death in all patients with ER+/HER2− mBC. Elacestrant is associated with a 45% reduction in the risk of progression or death in patients harbouring mESR1. Adverse events leading to elacestrant or standard of care discontinuation were rare in both arms (6.3% and 4.4%). The study conclusions were that Elacestrant is the first oral SERD to demonstrate statistically and clinically significant improvement in PFS versus standard of care endocrine therapy in a global phase 3 randomised study in postmenopausal men and women with ER+/HER2− mBC on second/third line post-CDK4/6i treatment and that clinically Elacestrant has the potential to become the new standard of care in the patient population studied and further analysis is awaited for final overall survival for next year.

The second study Dr Limon referred to was the KEYNOTE-522 [10] study of neoadjuvant pembrolizumab + chemotherapy versus placebo + chemotherapy, followed by adjuvant pembrolizumab versus placebo for early-stage TNBC: 1,714 patients were randomised 2:1, meeting key criteria of eligibility: Age ≥ 18 years, newly diagnosed TNBC of T1c N1-2 or T2-4 N0-2, ECOG PS 0–1, tissue sample for PD-L1 evaluation. Primary endpoints pCR (ypT0/Tis ypN0) assessed by Local Pathologist in the intention to treatment (ITT) Population, event free survival (EFS) assessed by Investigator in ITT Population, secondary endpoints pCR by alternative definitions (ypT0 ypN0 and ypT0/Tis), overall survival, pCR, EFS and overall survival in the PD-L1-positive population, safety in all treated patients. Exploratory analyses, sensitivity analyses of EFS and EFS in subgroups of patients, results were favourable for the pembrolizumab-associated chemotherapy combination compared to the control arm with an HR of 0.63 for event-free survival. Multivariate analysis showed that all subgroups benefited from the combination compared to the control arm. The study conclusions were: neoadjuvant pembrolizumab + chemotherapy followed by adjuvant pembrolizumab resulted in statistically and clinically significant improvement in EFS. Prespecified EFS sensitivity analyses show a strong benefit of treatment with neoadjuvant pembrolizumab + chemotherapy followed by adjuvant pembrolizumab for previously untreated nonmetastatic TNBC. This benefit was generally consistent across a wide selection of prespecified patient subgroups, including those defined by nodal status and overall disease stage. He tells us that the study shows that safety was consistent with the known profiles of each regimen. These results further support pembrolizumab plus neoadjuvant platinum-containing chemotherapy, followed by adjuvant pembrolizumab after surgery, as a new standard treatment regimen for high-risk early-stage TNBC patients.

## Conclusions

Definitely after the difficult years since the start of the COVID-19 pandemic, we are optimistic about the restart of academic activities since we are aware of the importance of continuing medical education. During the 2 days of the congress, we were able to talk and introduce new and updated information in different fields of oncology, focused for the first time on ‘choosing wisely’. This concept was developed for the first time in the United States and this congress format was applied for the first time in Latin America, where the main objective was to share updated information in addition to applying the ‘choosing wisely’ concept in the different topics of the event. This congress allowed speakers and attendees to share face to face, as we are aware that face-to-face interaction is very important for understanding the different topics presented. Finally, Dr Ronald Limon proceeded to close the congress concluding: choosing wisely is a concept that can be applied in all areas of oncology, and it is important to optimise our human, economic and material resources in order to offer adequate treatments with patients without overstepping therapeutic decisions [17, 18].

## Conflicts of interest and funding

The author is not aware of any affiliations, memberships, funding or financial holdings that might be perceived as affecting the objectivity of this review.
